# Neutrophil to lymphocyte ratio predicting poor outcome after aneurysmal subarachnoid hemorrhage: A retrospective study and updated meta-analysis

**DOI:** 10.3389/fimmu.2022.962760

**Published:** 2022-08-09

**Authors:** Yinghan Guo, Jiang Liu, Hanhai Zeng, Lingxin Cai, Tingting Wang, Xinyan Wu, Kaibo Yu, Yonghe Zheng, Huaijun Chen, Yucong Peng, Xiaobo Yu, Feng Yan, Shenglong Cao, Gao Chen

**Affiliations:** ^1^ Department of Neurosurgery, The Second Affiliated Hospital of Zhejiang University School of Medicine, Hangzhou, China; ^2^ Department of Neurosurgery, China-Japan Friendship Hospital, Beijing, China; ^3^ Department of Neurosurgery, First People’s Hospital of Jiashan County, Jiashan, China

**Keywords:** neutrophil to lymphocyte ratio, aneurysmal subarachnoid hemorrhage, poor outcome, delayed cerebral ischemia, retrospective study, meta-analysis

## Abstract

**Background:**

The relationship between neutrophil to lymphocyte ratio (NLR) and poor outcome of aneurysmal subarachnoid hemorrhage (aSAH) is controversial. We aim to evaluate the relationship between NLR on admission and the poor outcome after aSAH.

**Method:**

Part I: Retrospective analysis of aSAH patients in our center. Baseline characteristics of patients were collected and compared. Multivariate analysis was used to evaluate parameters independently related to poor outcome. Receiver operating characteristic (ROC) curve analysis was used to determine the best cut-off value of NLR. Part II: Systematic review and meta-analysis of relevant literature. Related literature was selected through the database. The pooled odds ratio (OR) and corresponding 95% confidence interval (CI) were calculated to evaluate the correlation between NLR and outcome measures.

**Results:**

Part I:** **A total of 240 patients with aSAH were enrolled, and 52 patients had a poor outcome. Patients with poor outcome at 3 months had a higher admission NLR, Hunt & Hess score, Barrow Neurological Institute (BNI) scale score, Subarachnoid Hemorrhage Early Brain Edema Score (SEBES), and proportion of hypertension history. After adjustment, NLR at admission remained an independent predictor of poor outcome in aSAH patients (OR 0.76, 95% CI 0.69-0.83; P < 0.001). The best cut-off value of NLR in ROC analysis is 12.03 (area under the curve 0.805, 95% CI 0.735 - 0.875; *P* < 0.001). Part II: A total of 16 literature were included. Pooled results showed that elevated NLR was significantly associated with poor outcome (OR 1.31, 95% CI 1.14-1.49; *P* < 0.0001) and delayed cerebral ischemia (DCI) occurrence (OR 1.32, 95% CI 1.11-1.56; *P* = 0.002). The results are more reliable in large sample sizes, low NLR cut-off value, multicenter, or prospective studies.

**Conclusion:**

Elevated NLR is an independent predictor of poor outcome and DCI occurrence in aSAH.

## Introduction

Aneurysmal subarachnoid hemorrhage (aSAH) is a serious cerebrovascular disease with high mortality and disability rate, accounting for about 25% of stroke deaths (1). Although high-quality craniotomy clipping, intravascular embolization, and intensive care have greatly reduced the incidence of death after aSAH in recent years, complications such as rebleeding, delayed cerebral ischemia (DCI), hydrocephalus still led to poor outcome in a large number of aSAH patients. In addition to actively preventing and treating these complications, preoperative accurate prediction of prognosis is very important because it can help clinicians to judge whether more active treatment selection and postoperative monitoring are needed.

Studies have shown that up to 60% of patients with hemorrhagic stroke, including aSAH, will develop systemic inflammatory response syndrome (SIRS) after onset, which can be characterized by increased peripheral neutrophils (2–5). At the same time, it has been reported that some patients with aSAH have immunosuppression characterized by lymphocytopenia (6). Neutrophil to lymphocyte ratio (NLR) is a novel marker of the systemic inflammatory response, characterized by easy acquisition and standardization. In recent years, various studies have reported that NLR can be used as a prognostic factor for cerebrovascular accidents such as ischemic cerebral infarction and cerebral hemorrhage. At the same time, there have been many reports about NLR and the poor outcome of aSAH patients. Studies have shown that elevated NLR at admission is associated with higher modified Rankin Scale (mRS) score 3 months after aSAH, suggesting that higher NLR can be a predictor of poor prognosis (2, 7, 8). Other studies have shown that elevated NLR is not significantly associated with poor outcome after aSAH (9–12).

Given this, this study aims to provide the experience of our center on this research topic and obtain more reliable evidence-based medical evidence through systematic review and meta-analysis of relevant literature. In addition, radiology markers related to poor outcome after aSAH [e.g., Barrow Neurological Institute (BNI) scale (13) and Subarachnoid Hemorrhage Early Brain Edema Score (SEBES) (14)] were also included to evaluate the predictive independence of NLR.

## Methods

### Part I retrospective study

#### Patient selection

Data for all consecutive patients with aSAH admitted from May 2018 to July 2020 to the Departments of Neurosurgery of the Second Affiliated Hospital of Zhejiang University were collected. Inclusion criteria: 1) Patients with aSAH confirmed by CT angiography or digital subtraction angiography who underwent surgery or interventional therapy; 2) age 18-80 years; 3) Patients who received treatment for ruptured aneurysms within 72 h; 4) Admission within 24 h of initial symptoms onset; 5) Initial blood sampling for laboratory test was performed within 24 h after bleeding. Exclusion criteria: 1) Patients with acute or chronic infection, cardiovascular and cerebrovascular diseases, hematological diseases, autoimmune diseases, and other systemic diseases (including but not limited to malignant tumors, uremia, and severe liver and kidney dysfunction); 2) Patients lost follow-up. The study was approved by the Hospital Ethics Committee of the Second Affiliated Hospital of Zhejiang University and informed consent of all patients or legal representatives.

#### Clinical variables and outcome

Demographic data (age, gender), past medical history (hypertension, diabetes), medication history (nimodipine), admission status (Hunt & Hess score, GCS score, and WFNS grade), neuroradiological data (intraventricular hemorrhage, mFisher grade, BNI scale (13), and SEBES (14)), treatment methods (craniotomy clipping or interventional embolization), laboratory indexes (neutrophils, lymphocytes, monocytes/macrophages, and NLR), and complications (rebleeding, DCI, hydrocephalus, intracranial infection, and pulmonary infection) were recorded. The NLR is calculated by dividing the absolute neutrophil count by the absolute lymphocyte count. The prognosis was assessed by the modified Rankin Scale (mRS) 3 months after aSAH through semistructured telephone interviews. mRS ≥ 3 was defined as a poor outcome.

### Statistics

Patients were classified according to prognosis at 3 months for analysis (mRS score 0–2 vs. mRS score 3–6). Categorical variables were expressed as numbers and percentages that were analyzed using chi-square or Fisher exact test. Continuous variables were presented as the mean ± standard deviation (SD) or median with interquartile range (IQR) and analyzed by Mann - Whitney U test. The independent predictors of aSAH prognosis were identified by univariate analysis and multivariate logistic regression analysis. The variables with *P* < 0.05 in univariate analysis were included in the multivariate logistic regression model. In the final multivariate model, the predictive variable *P* < 0.05 was considered to have a significant correlation. Furthermore, the correlation between NLR and aSAH poor outcome was evaluated by receiver operating characteristics curve (ROC), in which the best cut-off value for prognosis prediction was determined. Analyses were performed using SPSS 24.0 (IBM Corp., Armonk, NY, USA) and R 3.6.3 (R Foundation for Statistical Computing, Vienna, Austria).

### Part II meta-analysis

#### Search strategy

We conducted a comprehensive search of the English literature on February 27, 2022, using PubMed, Embase, Cochrane Library, and Web of Science databases, taking into account all available papers without limitation of publication time. Searched with the following keywords: (subarachnoid hemorrhage OR SAH OR ruptured brain aneurysm OR ruptured cerebral aneurysm) AND (neutrophil to lymphocyte ratio OR neutrophil-lymphocyte ratio OR neutrophil-to-lymphocyte ratio OR NLR). All selected articles were independently screened by two researchers (Guo and Zeng), and a consensus was reached on the differences that arose.

#### Selection criteria

Inclusion criteria: 1) Clinical study of adult aSAH patients (18-80 years old); 2) Articles reporting the correlation between NLR and aSAH, and the definition of poor prognosis and DCI were described below; 3) Multivariate analysis in the paper provides odds ratio (OR) or risk ratio (RR) and 95% confidence interval (CI) related to outcome.

Exclusion criteria: 1) Meta-analysis, reviews, letters, case reports, comments, and other unrelated basic studies; 2) Studies that only provide unadjusted univariate analysis data; 3) Incomplete or defective data; 4) English full text is not available.

#### Outcome measure

The two main prognostic indicators in the meta-analysis were poor outcome and occurrence of DCI. Poor outcome was defined as Glasgow Outcome Scale (GOS) score of 1 to 3 or an mRS score of 3 to 6 at the end of follow-up. The definition of DCI is as follows (15, 16): 1) Symptoms of permanent or temporary focal neurological dysfunction, such as hemiplegia, hemianopia, aphasia, apraxia, etc., occurred 4−14 days after aSAH, while no obvious hemorrhage, hematoma, hydrocephalus, etc. were found in head CT scan; 2) The Glasgow coma score decreased by at least 2 points and lasted at least 1 h, with no obvious symptoms at the time after surgery; 3) Head CT scan within 4-30 days after aSAH found new cerebral infarction that was not present at admission or at the time after surgery, which could not be explained by other reasons except vasospasm.

#### Data extraction and quality assessment

Relevant data including author, publication year, duration, country, case number, sample time, outcome measure, follow-up, cut-off value, and article type were collected. OR/RR values and 95% CI were extracted preferentially from multivariate analysis with NLR as a dichotomous variable, otherwise from multivariate analysis with NLR as a continuous variable. The quality of the included literature was assessed by the Newcastle-Ottawa Scale (NOS) score (17). The score includes three parts: cohort selection, comparability, and results. The total score ranges from 0 to 9, with the higher the score, the better the quality. Studies that score ≥6 were considered high quality.

#### Statistics

Data analysis was performed using RevMan 5.4 analysis software (Cochrane Collaboration, Copenhagen, Denmark). The extracted OR/RR values and 95% CI were weighted by the inverse variance method to evaluate the association of NLR with poor prognosis or the occurrence of DCI. Heterogeneity was assessed by *I^2^* and Q statistical tests. When *I^2^* > 50% or *P* < 0.05 was considered to have significant heterogeneity, a random-effects model was used for analysis, and when *I^2^* < 50% and *P* > 0.05 were considered to have no significant heterogeneity, a fixed-effects model was used. The results of the meta-analysis were shown by forest plot, and *P* < 0.05 was considered statistically significant. Sources of heterogeneity were identified by subgroup analysis. Publication bias was assessed by funnel plots.

## Result

### Part I retrospective study

#### Patient characteristic

**Figure 1:**
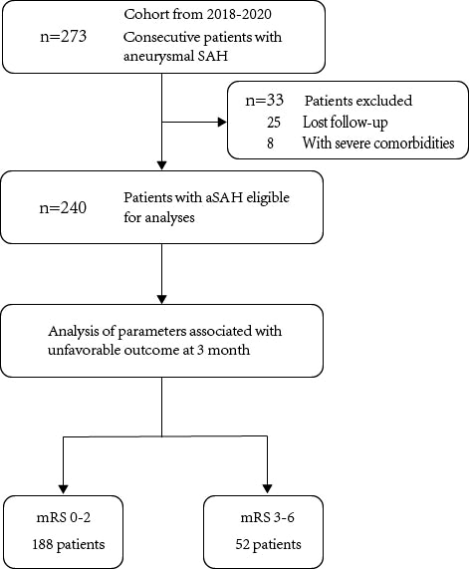
Flow diagram of patient selection.

A total of 273 aSAH patients were screened, among which 25 patients lost follow-up (9.16%) and eight patients had comorbidities such as malignant tumor and severe hepatic and renal dysfunction (2.93%). Finally, 240 patients were enrolled and divided into good prognosis group (mRS 0-2, n =188) and poor prognosis group (mRS 3-6, n = 52) according to mRS score at 3 months after aSAH (**Figure 1**). Univariate analysis showed that patients with poor prognosis at 3 months [mRS 3 - 6: 52/240 (21.7%)] had a higher proportion of hypertension history [79 (42.0%) for mRS 0 - 2 vs. 33 (63.5%) for mRS 3 - 6; *P* < 0.001], higher admitted Hunt & Hess score [median (IQR) 2 (1 – 3) for mRS 0 - 2 vs. 2.5 (2 – 3) for mRS 3 - 6; *P* < 0.001], higher BNI scale score [median (IQR) 5 (4 – 5) for mRS 0 - 2 vs. 5 (5 – 5) for mRS 3 - 6; *P* = 0.013], and higher SEBES [median (IQR) 1 (0 – 2) for mRS 0 - 2 vs. 2 (0 – 3) for mRS 3 - 6; *P* = 0.010]. In addition, these patients had significantly higher neutrophils count within 24 h of admission (mean ± SD 10.41 ± 3.88 for mRS 0 - 2 vs. 12.43 ± 3.66 for mRS 3 - 6; *P* = 0.001), significantly lower lymphocytes count [median (IQR) 1.04 (0.86 - 1.34) for mRS 0 – 2 vs. 0.78 (0.60 - 1.01) for mRS 3 - 6; *P* < 0.001], and significantly higher NLR ratio (mean ± SD 9.76 ± 3.98 for mRS 0 - 2 vs. 16.13 ± 6.20 for mRS 3 - 6; *P* < 0.001 **Table 1**).

**Table 1:** Patient characteristics.

Patient Characteristics (n = 240)	mRS score at 3 months			*P* value	
	0–2 (n = 188)	3–6 (n = 52)			
Age, yrs	55.93 ± 11.26	54.85 ± 11.88		0.544	
Female sex	119 (63.3%)	31 (59.6%)		0.627	
Prior medical history ** Hypertension** Diabetes Medication history Nimodipine	79 (42.0%) 5 (2.7%) 162 (86.2%)	33 (63.5%) 3 (5.8%) 52 (75%)		**0.006** 0.503 0.053	
Admission status ** Hunt–Hess** 1 2 3 4 5 GCS score 3-8 9-12 13-15 WFNS grade 1 2 3 4 5 Neuroradiological data Intraventricular hemorrhage mFisher scale ** BNI scale** ** SEBES**	76 (40.4%) 62 (33.0%) 26 (13.8%) 20 (10.6%) 4 (2.1%) 23 (12.2%) 4 (2.1%) 161 (85.6%) 141 (75.0%) 18 (9.6%) 2 (1.1%) 18 (9.6%) 9 (4.8%) 100 (53.2%) 3.00 (2.00-4.00) 5.00 (4.00-5.00) 1.00 (0.00-2.00)	5 (9.6%) 21 (40.4%) 20 (38.5%) 6 (11.5%) 0 (0.0%) 10 (19.2%) 2 (3.8%) 40 (76.9%) 33 (63.5%) 6 (11.5%) 1 (1.9%) 6 (11.5%) 6 (11.5%) 32 (61.5%) 3.00 (2.00-4.00) 5.00 (5.00-5.00) 2.00 (0.00-3.00)		**<0.001** 0.317 0.267 0.284 0.510 **0.013** **0.010**	
Endovascular coiling	71 (37.8%)	20 (38.5%)		0.927	
Lab values on admission ** NLR** ** Neutrophils, ×10^9^/L** ** Lymphocytes, ×10^9^/L** Monocytes, ×10^9^/L Complications Rebleeding ** DCI** Hydrocephalus Intracranial infection Pulmonary infection	9.76 ± 3.98 10.41 ± 3.88 1.04 (0.86-1.34) 0.45 (0.29-0.65) 7 (3.7%) **7 (3.7%)** 7 (3.7%) 17 (9.0%) 20 (10.6%)	16.13 ± 6.20 12.43 ± 3.66 0.78 (0.60-1.01) 0.47 (0.25-0.72) 3 (5.8%) **14 (26.9%)** 5 (9.6%) 6 (11.5%) 10 (19.2%)		**<0.001** **0.001** **<0.001** 0.973 0.794 **<0.001** 0.172 0.783 0.097	

The bold values were considered statistically significant.

#### Relationship between NLR and functional outcome

To identify the independent predictors associated with 3 months poor outcome, variables with *P* < 0.05 in univariate analysis were included in the multivariable logistic regression model. Since neutrophils and lymphocytes were associated with NLR, they were not included in the multivariable logistic regression model. After adjustment, high NLR at admission was independently associated with poor prognosis in aSAH patients (OR 0.760, 95% CI 0.693-0.833; *P* < 0.001). In addition, previous history of hypertension (OR 0.409, 95% CI 0.191-0.876; *P* = 0.021) and high SEBES (OR 0.753, 95% CI 0.573-0.991; *P* = 0.043, **Table 2**) were also independently associated with poor prognosis.

**Table 2:** Multivariate analysis of parameters associated with poor outcome at 3 months.

Parameter	OR (95% CI)	*P* Value
**NLR**	0.760 (0.693-0.833)	**<0.001**
**Hypertension** **SEBES**	0.409 (0.191-0.876) 0.753 (0.573-0.991)	**0.021** **0.043**

The bold values were considered statistically significant.

**Figure 2:**
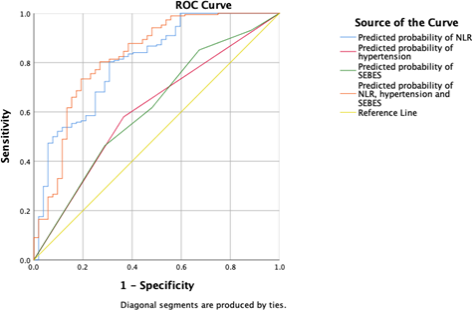
ROC analysis of the correlation between NLR and mRS at 3 months.

In the ROC curve (**Figure 2**), NLR of 12.03 was identified as the best cut-off value for distinguishing 3 months favorable and unfavorable outcome [area under the curve (AUC) 0.805, 95% CI 0.735 - 0.875; P<0.001].

### Part II meta-analysis

#### Study selection

**Figure 3:**
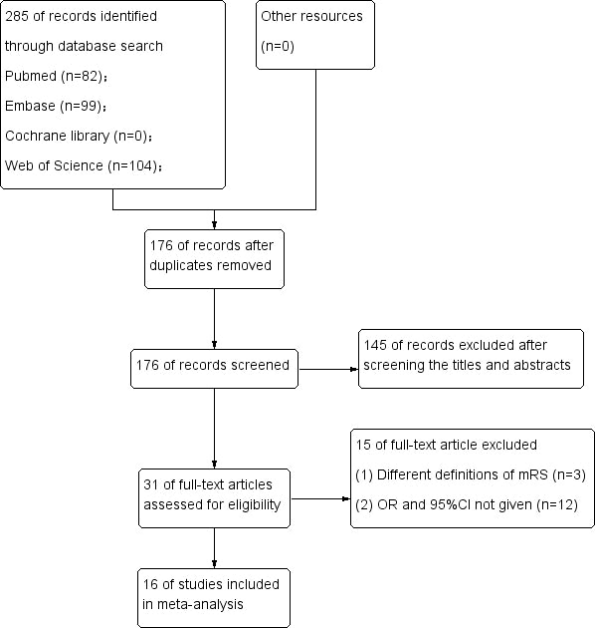
Flow diagram of study selection.

A total of 285 articles were screened (82 in PubMed, 99 in Embase, 0 in Cochrane Library, and 104 in Web of Science), with 176 articles remained after eliminating duplicates. Moreover, 145 studies unrelated to “aSAH” or “NLR” were excluded through a quick browse of titles and abstracts. In addition, 15 articles were excluded according to the inclusion and exclusion criteria after full text read of the remaining 31 articles. Among them, the mRS definitions of 3 articles were different from those mentioned above, 12 articles did not provide OR and 95%CI, and 16 articles that met the criteria were finally included (**Figure 3**).

#### Study characteristics

In total, we included 16 articles in this meta-analysis (**Table 3**), published from 2016 to 2022, and studied from 2004 to 2021. 10 studies were conducted in Asia (7 in China, 2 in Korea, and 1 in the Philippines), 2 in Europe (1 in Germany, 1 in Belgium), and 4 in the United States. Most of them were single-center studies (13 single-center studies, 3 multi-center studies) and retrospective studies (13 retrospective studies, 3 prospective studies). A total of 6180 aSAH patients were enrolled, who generally had blood test results between admission and day 5. The prognosis and occurrence of DCI were followed up from discharge to 6 months, and the optimal cut-off value of NLR ranged from 4 to 14. 14 of the original studies were of high quality with NOS score ≥6, and 2 conference abstracts had NOS score less than 6.

**Table 3:** Baseline characteristic of the enrolled studies.

Studies	Duration	Country	Research center	Design	Number	Sample time (within)	Outcome measure	Follow-up	Cut-off value	NOS	Article type
Al-Mufti 2017 (18)	/	USA	/	P	849	72h	DCI	/	10.75	5	CA
Al-Mufti 2019 (9)	2006–2015	USA	S	P	1067	24h	mRS/DCI	3 Mos	5.9	8	OR
Chang 2021 (19)	2015-2019	USA	M	R	474	Adm	mRS	Disc	6.48	8	OR
Chen 2020 (11)	2015–2019	CN	S	R	262	Adm	mRS	3 Mos	/	8	OR
Geraghty 2021 (20)	2013-2019	USA	S	R	246	24h	DCI	/	/	7	OR
Giede-Jeppe 2019 (2)	2008–2012	GM	S	R	319	Adm	mRS	12 Mos	7.05	8	OR
Groza 2016 (21)	2004-2015	BE	S	R	123	5 days	DCI	/	/	5	CA
Hu 2021 (22)	2019-2020	CN	S	R	126	/	mRS	3 Mos	/	7	OR
Ignacio 2022 (23)	2015-2020	PH	S	R	222	24h	mRS/DCI	Disc	/	7	OR
Lai 2020 (7)	2016–2018	CN	S	R	235	/	mRS	3 Mos	/	8	OR
Tao 2017 (8)	2014–2015	CN	S	P	247	Adm	mRS/DCI	3 Mos	14	8	OR
Wu 2019 (5)	2015, 1–12	CN	S	R	122	Adm	DCI	Disc	11.47	7	OR
Yi 2020 (24)	2012–2020	KR	M	R	498	Adm	mRS/DCI	3 Mos	5.7	8	OR
Yun 2021 (10)	2012–2021	KR	M	R	680	Adm	mRS	3 Mos	4.0	8	OR
Zhang 2020 (12)	2015–2017	CN	S	R	178	Adm	GOS	3 Mos	/	7	OR
Zhang 2021 (25)	2013–2016	CN	S	R	532	Adm	mRS/DCI	3 Mos	4.0	7	OR

P, Prospective research; R, retrospective; S, Single-center; M, Multi-center; CA, Conference Abstract; OR, Original Research; CN, China; KR, Korea; PH, Philippines; BE, Belgium; GM, German; Disc, discharge; Mos, months.

#### Association of NLR with poor outcome

**Figure 4:**
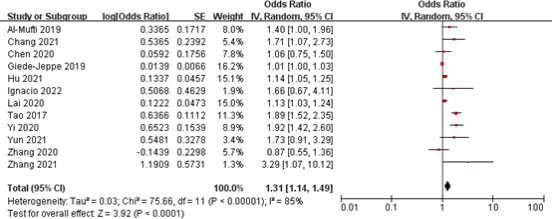
Meta-analysis of NLR and poor outcome.

Twelve studies involving a total of 4,840 patients assessed the association of NLR with poor outcome after aSAH. Pooled OR suggested that elevated NLR after admission was significantly associated with poor outcome after aSAH (OR 1.31, 95% CI 1.14-1.49; *P* < 0.0001), with significant heterogeneity (*I^2^* = 85%, *P* < 0.00001) (**Figure 4**).

#### Association of NLR with DCI occurrence

**Figure 5:**
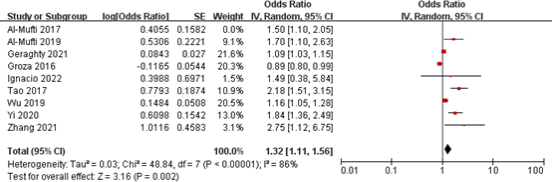
Meta-analysis of NLR and DCI occurrence.

Nine studies involving a total of 3,906 patients assessed the association between NLR and DCI occurrence after aSAH. Pooled OR suggested that elevated NLR after admission was significantly associated with DCI occurrence after aSAH (OR 1.32, 95% CI 1.11-1.56; *P* = 0.002), with significant heterogeneity (*I^2^* = 86%, *P* < 0.00001) (**Figure 5**).

#### Subgroup analysis

It was assumed that different ethnicity, sample size, study design, cut-off value, research center, and NOS score in different studies would affect the correlation between NLR and poor outcome or DCI occurrence. We divided the different studies into six subgroups for analysis. Since NOS of the poor outcome-related articles were all ≥6, the NOS score subgroup was not set. The DCI occurrence-related articles only involved one multi-center study, so there was no subgroup of research centers (**Table 4**).

**Table 4:** Subgroup analysis of NLR with poor outcome and DCI occurrence.

Subgroup	Number	model	*I^2^*	*P* value	OR (95% CI)	*P* value
Poor outcome	12	R	85%	<0.00001	1.31[1.14, 1.49]	<0.0001
Ethnicity						
Asian	9	R	78%	<0.0001	1.35 [1.14, 1.62]	0.0007
Non-Asian	3	R	76%	0.02	1.27 [0.91, 1.76]	0.16
Sample size						
≥400	**5**	**R**	**0%**	**0.52**	**1.71 [1.42, 2.07]**	**<0.00001**
<400	7	R	86%	<0.00001	1.17 [1.03, 1.34]	0.02
Study design						
Retrospective	10	R	78%	<0.00001	1.20 [1.06, 1.35]	0.003
Prospective	2	R	54%	0.14	1.67 [1.25, 2.23]	0.0005
Cut-off value						
<7	**5**	**R**	**0%**	**0.52**	**1.71 [1.42, 2.07]**	**<0.00001**
≥7	2	R	97%	<0.00001	1.37 [0.74, 2.52]	0.31
Research center						
Multi-center	**3**	**R**	**0%**	**0.90**	**1.84 [1.45, 2.33]**	**<0.00001**
Single-center	9	R	85%	<0.00001	1.21 [1.06, 1.37]	0.005
DCI	9	R	86%	<0.00001	1.32 [1.11, 1.56]	0.002
Ethnicity						
Asian	5	R	80%	0.0005	1.72 [1.17, 2.51]	0.005
Non-Asian	4	R	85%	0.0001	1.15 [0.94, 1.39]	0.17
Sample size						
≥400	**4**	**R**	**0%**	**0.58**	**1.71 [1.41, 2.07]**	**<0.00001**
<400	5	R	86%	<0.00001	1.15 [0.97, 1.35]	0.10
Study design						
Retrospective	7	R	83%	<0.00001	1.21 [1.04, 1.41]	0.02
Prospective	**2**	**R**	**0%**	**0.39**	**1.97 [1.48, 2.60]**	**<0.00001**
Cut-off value						
<7	**3**	**R**	**0%**	**0.64**	**1.85 [1.46, 2.35]**	**<0.00001**
≥7	3	R	84%	0.002	1.51 [1.05, 2.17]	0.03
NOS						
<6	2	R	90%	0.002	1.13 [0.68, 1.88]	0.64
≥6	7	R	81%	<0.0001	1.45 [1.20, 1.76]	0.0001

The bold values were considered statistically significant.

**Figure 6:**
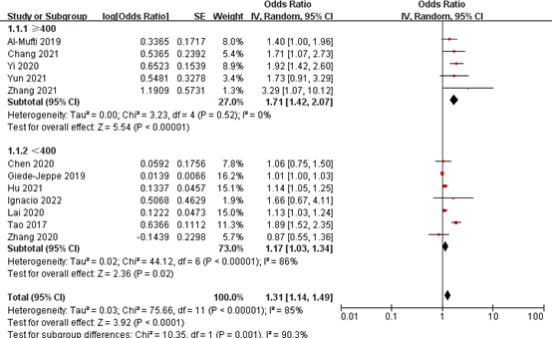
Subgroup analysis of NLR and poor outcome (sample size group).

In the subgroup analysis of NLR and poor outcome, we found no significant heterogeneity in the pooled results within three subgroups (**Table 4**). In the subgroup with sample size of ≥400, NLR was significantly associated with poor outcome (OR 1.71, 95% CI 1.42-2.07; *P* < 0.00001), with no significant heterogeneity (*I^2^* = 0%, *P* = 0.52). There was also a significant correlation between them in the subgroup with a sample size of <400 (OR 1.17, 95% CI 1.03-1.34; *P* = 0.02); however, there was a significant heterogeneity in the pooled results (*I^2^* = 86%, *P* < 0.00001) (**Figure 6**). In the subgroup with a cut-off value of <7, patients with higher NLR were 1.71-fold more likely to have poor outcome than those with lower NLR (OR 1.71, 95% CI 1.42-2.07; *P* < 0.00001), the combined results were reliable (*I^2 =^* 0%, *P* = 0.52). However, there was no significant correlation between them in the subgroup with a cut-off value of ≥7 (OR 1.37, 95% CI 0.74-2.52; *P* = 0.31). In the multicenter subgroup, higher NLR was able to predict poor outcome (OR 1.84, 95% CI 1.45-2.33; *P* < 0.00001), with no significant heterogeneity (*I^2^* = 0%, *P* = 0.90). The similar results were found in the single-center subgroup (OR 1.21, 95% CI 1.06-1.37; *P* = 0.005); however, the pooled results were less reliable (*I^2^ =*85%, *P* < 0.00001).

**Figure 7:**
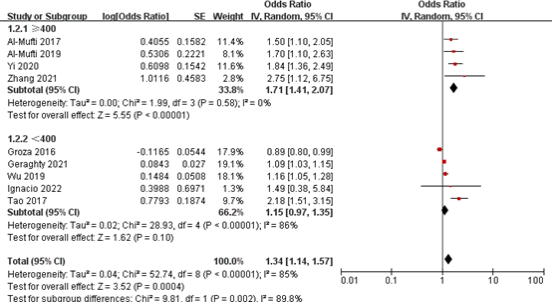
Subgroup analysis of NLR and DCI occurrence (sample size group).

For the subgroup analysis of NLR and DCI occurrence, we also found that there was no significant heterogeneity in the pooled results within three subgroups (**Table 4**). In the subgroup with a sample size of ≥400, NLR was significantly associated with the occurrence of DCI (OR 1.71, 95% CI 1.41-2.07; *P* < 0.00001), with no significant heterogeneity (*I^2^* = 0%, *P* = 0.58). In the subgroup with a sample size of <400, there was no significant correlation between them (OR 1.15, 95% CI 0.97-1.35; *P* = 0.10) (**Figure 7**). In the subgroup with a cut-off value of <7, patients with higher NLR had a 1.85-fold risk of DCI occurrence than patients with lower NLR (OR 1.85, 95% CI 1.46-2.35; *P* < 0.00001), and the pooled results were reliable (*I^2^* = 0%, *P* = 0.64). There was also a significant association between them in the subgroup with a cut-off value of ≥7 (OR 1.51 95% CI 1.05-2.17; *P* = 0.03); however, the pooled results were unreliable (*I^2^* = 84%, *P* = 0.002). In the prospective subgroup, higher NLR was able to predict the occurrence of DCI (OR 1.97, 95% CI 1.48-2.60; *P* < 0.00001), with no significant heterogeneity (*I^2^* = 0%, *P* = 0.39). There was also a significant association between them in the retrospective subgroup (OR 1.51 95% CI 1.05, 2.17; *P* = 0.03); however, the pooled results were less reliable (*I^2^* = 83%, *P* < 0.00001).

#### Publication bias

**Figure 8:**
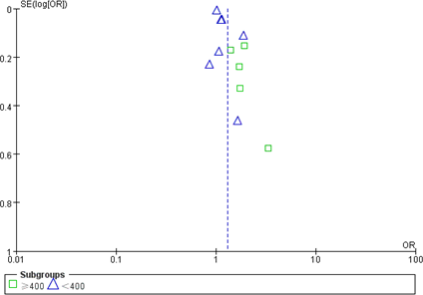
Funnel plot of publication bias of NLR with poor outcome.

**Figure 9:**
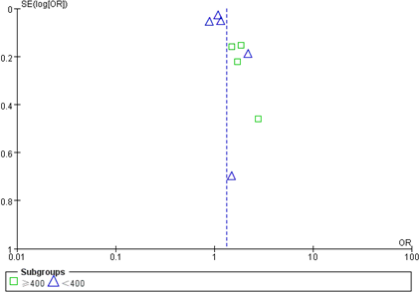
Funnel plot of publication bias of NLR with DCI occurrence.

Two funnel plots were made to evaluate publication bias, and no obvious publication bias was observed (**Figures 8**, **9**).

## Discussion

First, in our single-center retrospective study, we confirmed that elevated NLR at admission was significantly correlated with poor outcome at 3 months after aSAH, and the best predictive value of NLR was 12.03. Compared with previous studies, we included BNI scale and SEBES for the first time, further demonstrating the predictive independence of NLR. In addition, we systematically assessed the relationship between NLR and poor outcome and DCI occurrence through meta-analysis. The pooled results showed that higher NLR predicted poor outcome and DCI occurrence and the pooled results were highly heterogeneous. Although further subgroup analysis did not reveal a clear source of heterogeneity, we found that the pooled results in many subgroups had low heterogeneity. We believe that elevated NLR is more reliably associated with poor outcome and DCI occurrence in studies with large sample sizes, low cut-off value, multicenter, or prospective studies. In conclusion, NLR may be a new independent predictor of poor prognosis in aSAH, and our findings provide relatively reliable evidence for evidence-based medicine.

Why is the elevated NLR associated with the 3 months poor outcome of aSAH? First, we need to be clear that the increase of NLR includes two parts: excessive neuroinflammation indicated by neutrophil increase and immunosuppressive state indicated by lymphocyte decrease. On the one hand, neutrophil infiltration is involved in early brain injury after aSAH, and peripheral blood NLR may reflect the severity of neutrophil infiltration after aSAH to a certain extent. Basic research shows that neutrophils can aggregate and adhere to microvascular endothelium 10 min after bleeding (26), and gradually infiltrate the damaged local brain parenchyma under the action of chemokines. Excessive accumulation of neutrophils can damage the brain parenchyma by releasing reactive oxygen species, cathepsin, matrix metalloproteinases, neutrophil extracellular traps (NETs), etc. (27), and they can interact with resident brain inflammatory cells to aggravate the progression of neuroinflammation. Studies had shown that higher NLR levels were associated with more severe nerve injury, as reflected in more severe subarachnoid hemorrhage (modified Fisher Scale grade) and intraventricular hemorrhage in patients with NLR > 7.05 (2). It has also been shown that the higher the NLR, the lower the Glasgow coma score at admission (28). Since clinical severity of aSAH is a major predictor of adverse outcome, NLR may indirectly reflect a poor outcome by reflecting clinical severity. On the other hand, lymphocyte depletion after aSAH suggests a post bleeding immunosuppression, which may lead to infectious complications. Studies have reported that some patients with SAH suffered from immunosuppression accompanied by lymphocytopenia (6). The specific mechanism is unknown, which may be related to the activation of the sympathetic nervous system and hypothalamic pituitary adrenal axis caused by the physiological stress response, resulting in the release of endogenous stress mediators (catecholamines and glucocorticoids) (6). Studies have shown that patients with elevated NLR have a higher incidence of in-hospital pneumonia, ventriculitis, and urinary tract infection (2, 29, 30), which may prolong bedtime in aSAH patients, especially among the elderly, leading to the poor outcome at 3 months (30). Different studies have also found that NLR can be a reliable predictor of the prognosis of patients with cerebral infarction and intracerebral hemorrhage (31–34), which may suggest the common mechanism of secondary brain injury after these acute cerebrovascular diseases, that is, excessive inflammatory and immune imbalance. Therefore, regulating inflammation and immune status by targeting specific subsets of neutrophils or lymphocytes may be a potential therapeutic target.

Different studies have also reported that NLR is associated with other complications of aSAH, such as rebleeding and DCI, which may relate to unfavorable prognosis. Rebleeding usually occurs within 72 h after aSAH, and the association between NLR and rebleeding may lie in the accumulation of neutrophils that alter the wall stability of ruptured aneurysm (35). The clinical data of Wang et al. showed that NLR was an independent risk factor for rebleeding, and there was no doubt that patients with rebleeding had a higher proportion of poor outcome at 3 months (36). In addition, admission NLR is also associated with DCI after aSAH. Neutrophils in cerebrospinal fluid after hemorrhage may cause cerebral vasospasm (37), leading to DCI. In addition, excessive accumulation of neutrophils in microvessels not only causes vascular injury by releasing inflammatory mediators but also causes the formation of microvascular emboli, resulting in reduced cerebral blood perfusion (26, 38). Wu et al. found that NLR was negatively correlated with mean cerebral blood flow (mCBF) in brain CT perfusion scan of aSAH patients, and positively correlated with mean transit time (MTT) and mean time to peak (mTTP), suggesting slow and insufficient cerebral blood flow (5). Many studies have reported that elevated NLR can predict DCI occurrence after aSAH (9, 22, 23), whereas some studies have denied the correlation between them (19), so we conducted a meta-analysis of relevant literature. As mentioned above, our results show that elevated NLR is significantly correlated with the DCI occurrence after aSAH. Although rebleeding and DCI are early complications of aSAH, their occurrence often leads to a longer hospital stay and a slower recovery process, which indirectly leads to the poor outcome at 3 months.

As a readily available biochemical indicator, assessing admission NLR for aSAH patients can assist clinicians in making more appropriate treatment decisions and more active hospital monitoring. By combining NLR, Hunt & Hess score, World Federation of Neurosurgical Societies grade, modified Fisher Scale grade, and other indicators, clinicians can make a preliminary judgment of the severity and prognosis of a newly admitted aSAH patient. For patients with higher NLR, more rigorous intensive care treatment, more effective monitoring of postoperative complications, and more active follow-up after discharge should be carried out to reduce the proportion of adverse prognoses of aSAH patients. Given that NLR may be associated with immunosuppressive status, it is also worth discussing whether NLR can help us identify patients requiring additional treatment, such as whether patients with high NLR suggest the need for prophylactic antibiotics or iatrogenic immunoregulation to prevent infection. It is worth mentioning that related randomized controlled studies have been carried out in ischemic stroke (39, 40). Current evidence suggests that prophylactic use of ceftriaxone does not improve the 3 months mRS score in patients with ischemic stroke (39). In addition, other inflammatory biomarkers that have been already involved in the pathophysiology of stroke, for example, CRP, metalloproteinases, or interleukins, may be correlated with the poor outcomes of aSAH (41–44). It is also meaningful to establish a laboratory indicator related prognostic model by combining NLR with these inflammatory biomarkers. Although our study provides reliable evidence-based medical evidence, a more comprehensive evaluation is needed to achieve the clinical application of NLR. For example, some aSAH patients with comorbidity such as hypertension and diabetes may have elevated basal NLR, thus, affecting the accuracy of NLR prediction. In addition, most of the current studies collected single NLR values at admission or within 24 h, it is also necessary to evaluate the relationship between dynamic changes of NLR and prognosis after aSAH.

Our study has several limitations. First of all, there were fewer patients with poor outcome in our center, and propensity score matching was not conducted to correct confounding factors, which may affect the statistical results. Secondly, due to the limitations of research conditions, this study inevitably fell into the swamp of single-center, small sample size, and retrospective, leading to the possibility of systematic bias. In addition, although data from different articles confirm that NLR can be used as an independent predictor of poor outcome in aSAH patients, it is still challenging to determine the optimal predictive cut-off value of NLR. Different articles have different definitions of “high NLR” that may be related to ethnic differences and pre-existing comorbidities. Finally, no clear source of heterogeneity was found in our meta-analysis that weakened the credibility of the pooled results.

## Conclusion

Elevated NLR at admission was associated with poor outcome at 3 months of aSAH, with a best predictive reference value of 12.03 at our medical center. In addition, the pooled results suggest that elevated NLR was able to predict poor outcome and DCI occurrence. However, due to the heterogeneity of different studies, multicenter, large sample size, and prospective studies are warranted to obtain a more reliable cut-off value.

## Data availability statement

The original contributions presented in the study are included in the article/**Supplementary Material**. Further inquiries can be directed to the corresponding author.

## Ethics statement

The studies involving human participants were reviewed and approved by the Hospital Ethics Committee of the Second Affiliated Hospital of Zhejiang University. The patients/participants provided their written informed consent to participate in this study. Written informed consent was obtained from the individual(s) for the publication of any potentially identifiable images or data included in this article.

## Author contributions

All authors contributed to the article and approved the submitted version.

## Funding

This work was supported by the National Natural Science Foundation of China (Grant nos. 81971099, 81870908, 82171275, 82171273), and Natural Science Foundation of Xinjiang Uygur Autonomous Region (2019D01C094).

## Conflict of interest

The authors declare that the research was conducted in the absence of any commercial or financial relationships that could be construed as a potential conflict of interest.

## Publisher’s note

All claims expressed in this article are solely those of the authors and do not necessarily represent those of their affiliated organizations, or those of the publisher, the editors and the reviewers. Any product that may be evaluated in this article, or claim that may be made by its manufacturer, is not guaranteed or endorsed by the publisher.
